# Reassessment of clinical variables in cardiac resynchronization defibrillator patients at the time of first replacement: Death after replacement of CRT (DARC) score

**DOI:** 10.1111/jce.15031

**Published:** 2021-04-30

**Authors:** Dominic A. M. J. Theuns, Kaijbar Niazi, Beat A. Schaer, Christian Sticherling, Sing‐Chien Yap, Kadir Caliskan

**Affiliations:** ^1^ Department of Cardiology Erasmus MC Rotterdam The Netherlands; ^2^ Department of Cardiology University of Basel Hospital Basel Switzerland

**Keywords:** cardiac resynchronization therapy, comorbidity, implantable cardioverter‐defibrillator, mortality, primary prevention

## Abstract

**Introduction:**

Cardiac resynchronization defibrillator (CRT‐D) as primary prevention is known to reduce mortality. At the time of replacement, higher age and comorbidities may attenuate the benefit of implantable cardioverter‐defibrillator (ICD) therapy. The purpose of this study was to evaluate the progression of comorbidities after implantation and their association with mortality following CRT‐D generator replacement. In addition, a risk score was developed to identify patients at high risk for mortality after replacement.

**Methods and Results:**

We identified patients implanted with a primary prevention CRT‐D (*n* = 648) who subsequently underwent elective generator replacement (*n* = 218) from two prospective ICD registries. The cohort consisted of 218 patients (median age: 70 years, male gender: 73%, mean left ventricular ejection fraction [LVEF]: 36 ± 11% at replacement). Median follow‐up after the replacement was 4.2 years during which 64 patients (29%) died and 11 patients (5%) received appropriate ICD shocks. An increase in comorbidities was observed in 77 patients (35%). The 5‐year mortality rate was 41% in patients with ≥2 comorbidities at the time of replacement. A risk score incorporating age, gender, LVEF, atrial fibrillation, anemia, chronic kidney disease, and history of appropriate ICD shocks at time of replacement accurately predicted 5‐year mortality (*C*‐statistic 0.829). Patients with a risk score of greater than 2.5 had excess mortality at 5‐year postreplacement compared with patients with a risk score less than 1.5 (57% vs. 6%; *p* < .001).

**Conclusion:**

A simple risk score accurately predicts 5‐year mortality after replacement in CRT‐D patients, as patients with a risk score of greater than 2.5 are at high risk of dying despite ICD protection.

## 1 INTRODUCTION

Cardiac resynchronization therapy (CRT) is part of the standard management in selected patients with chronic heart failure (HF), reduced left ventricular ejection fraction (LVEF), and electrical dyssynchrony.^1^, ^2^ The combination with a defibrillator, that is, cardiac resynchronization defibrillator (CRT‐D), is supported by the lower sudden cardiac death rate due to defibrillator therapy in patients with HF and left ventricular dysfunction. Several years after the initial implantation, elective device replacement will be necessary because of battery depletion. However, at this point in time, some patients may face a limited prognosis due to advanced age and multiple comorbidities negating the benefit of defibrillator therapy. On the other hand, at least 20% of patients experienced appropriate shocks from their device before replacement.^3^ Appropriate implantable cardioverter‐defibrillator (ICD) shocks are associated with a subsequent 3–5‐fold increased risk of death among patients with primary prevention ICDs.^3^ Whether this association can be extended to survival postreplacement is unknown. Current guidelines for initial implantation state that patients should be expected to survive at least 1 year, but the issue of replacement is rarely covered. Recently, there is more debate to change this attitude also toward ICD replacement in every patient.^4^, ^5^ However, the paucity of data describing the characteristics and outcomes of patients receiving ICD replacements is a barrier to risk stratification and prediction and explains partly the lack of clear indications for replacement in practice guidelines. A few studies with heterogeneous study populations evaluated mortality and risk factors following device replacement.^6^, ^7^, ^8^, ^9^ The REPLACE registry included patients with pacemakers, ICDs, and CRT‐Ds.^10^ The study by Wuest et al.^8^ included primary and secondary prevention patients implanted with ICDs and CRT‐Ds. However, data in the setting of primary prevention patients who underwent CRT‐D replacement are not available. Therefore, the objectives of the current study were to evaluate the progression of comorbidities in a cohort of primary prevention patients with a CRT‐D between initial implant and replacement, their association with mortality, and to develop a mortality risk tool, designated as the death after replacement of CRT‐D (DARC) risk score.

## 2 METHODS

### 2.1 Study population

Patients for this retrospective observational cohort study were obtained from two prospective ICD registries of the cardiology departments of Erasmus MC and the University Hospital of Basel. In these registries, we identified all patients in whom a CRT‐D was implanted for chronic HF and primary prevention of sudden cardiac death between January 2005 to December 2017. In both cohorts, CRT implantation was indicated by symptomatic HF despite optimal medical therapy, an impaired LVEF (≤35%), and the presence of an inter‐ or intraventricular conduction delay (QRS duration ≥ 130 ms). For the purpose of the study, the cohort comprised only those patients who underwent generator replacement. The date of replacement served as the index date (“time zero”) for the analysis. The administrative censoring date for analyses was set at the end of December 2018 for all patients alive until that date. Over the years, indications for CRT and the programming of devices have changed. To identify possible trends, we defined three groups according to the implant year (1, 2005–2009; 2, 2010–2014; 3, 2015–2017).

The study protocol was approved by the Institutional Review Board of the Erasmus MC (MEC 2018‐1713) and the University Hospital of Basel (BASEC 2018‐329). This retrospective study was not subjected to the Dutch Medical Research Involving Human Subjects Act and the need for written informed consent was waived. The study was carried out according to the ethical principles for medical research involving human subjects established by the Declaration of Helsinki. The privacy of all patients and the confidentiality of their personal information were protected.

### 2.2 Clinical variables, comorbidities, and drug treatment

Baseline data on clinical variables, comorbidities, laboratory values, and drug treatment are prospectively collected in both ICD registries. Those parameters were reassessed at the time of the first generator replacement. Both ICD registries and medical records were reviewed to obtain data on these parameters at baseline and replacement. For the current study, we investigated the progression of non‐ICD indication‐related comorbidities between implantation and first elective replacement, and their association with mortality postreplacement. Non‐ICD indication‐related comorbidities were defined as atrial fibrillation (AF), diabetes mellitus, anemia, chronic obstructive pulmonary disease (COPD), peripheral vascular disease (PVD), cerebrovascular disease, cancer, and chronic kidney disease (CKD).

Diabetes mellitus was defined as HbA_1c_ > 6.5% or the use of oral hypoglycemic agents or the use of insulin; anemia as a serum hemoglobin concentration of less than 12 g/dl (female) or less than 13 g/dl (male). The renal function was assessed by estimating the glomerular filtration rate (eGFR) using the Chronic Kidney Disease Epidemiology Collaboration equation.^11^ Renal function was stratified into stages for CKD according to the KDIGO 2012 practice guideline: Stage 1, eGFR ≥ 90 ml/min/1.73 m^2^; Stage 2, eGFR: 60–89 ml/min/1.73 m^2^; Stage 3A, eGFR: 45–59 ml/min/1.73 m^2^; Stage 3B, 30–44 ml/min/1.73 m^2^; Stage 4, eGFR: 15–29 ml/min/1.73 m^2^; and Stage 5, eGFR less than 15 ml/min/1.73 m^2^.^12^ The presence of CKD was defined as an eGFR less than 60 ml/min/1.73 m^2^ according to the practice guidelines.

### 2.3 Follow‐up and ICD therapy event analysis

Follow‐up started at the time of ICD implantation. Device interrogation was performed on scheduled regular visits and after symptomatic events. At each visit, arrhythmic events with stored electrograms (EGMs) were retrieved from the device's memory. Appropriate ICD therapy was defined as antitachycardia pacing (ATP) or shock delivered for ventricular tachyarrhythmia; ventricular fibrillation (VF) or ventricular tachycardia (VT). The presence of atrioventricular dissociation (ventricular rate greater than atrial rate) was used to diagnose ventricular tachyarrhythmia when the baseline atrial rhythm is sinus rhythm. In the case of AF baseline atrial rhythm, ventricular tachyarrhythmias were defined as events with a sudden increase in rate combined with a change in the ventricular near‐field and far‐field EGM morphology from the baseline rhythm without biventricular pacing.

### 2.4 Endpoint

The clinical endpoint for this study was all‐cause mortality after replacement; patients who underwent cardiac transplantation or who received a ventricular assist device were censored on the day of surgery. The secondary endpoint was the association of appropriate ICD shock within the VF zone (being “potentially life‐threatening”) and mortality. In addition, the association of appropriate ATP within the VT zone and mortality were also evaluated.

### 2.5 Statistical analysis

The normality of distribution was assessed by using the Shapiro–Wilk test. Continuous variables are presented as mean ± *SD* or as median with 25th and 75th percentiles, where appropriate. Data were compared by the paired Student's *t* test or Mann–Whitney *U* test, as appropriate. Categorical data are expressed as percentages and compared with the McNemar test. The mortality rate was calculated using the Kaplan–Meier method and differences between the groups were evaluated by the log‐rank test. Univariate logistic regression analyses were used to determine potential clinical predictors of mortality, with the calculation of odds ratio (OR) with 95% confidence intervals (CIs). Any variable with a *p *< .10 was included as a covariate in a multivariate binary logistic regression model. The goodness of fit was evaluated by calculating the likelihood ratio (LR), Akaike information criterion (AIC), and Bayesian information criterion (BIC). A higher LR and lower AIC and BIC suggest better goodness of fit. For assessment of the performance of the model, discriminative ability and calibration are essential. Model discrimination was assessed by the use of Harrell *C*‐statistic and receiver operating characteristic area under the curve (ROC AUC). Discrimination was deemed poor if the *C*‐statistic was between 0.50 and 0.70, modest between 0.70 and 0.80, and good if ≥0.80. Model calibration was visualized by plotting the predicted risks against the observed risks in a calibration‐in‐the‐large plot stratified by five equal groups of ascending prediction probability. Finally, the prediction score was internally validated by performing bootstrap analysis of 1000 samples. A *p *< .05 was considered significant. All statistical analyses were performed using SPSS, version 24 (IBM Corp), and STATA, version 16.1 (Stata Corp).

## 3 RESULTS

During the study period, a total of 648 patients received a CRT‐D for the primary prevention of sudden cardiac death. Of these, 218 underwent at least one replacement and comprised the study cohort. The study population was predominantly male (73%) with a median age of 65 years (58–71 years) at implantation and 70 years (62–75 years) at replacement. Clinical characteristics at baseline and at the moment of first replacement are shown in Table 1. The mean time between implantation and generator replacement was 5.0 ± 1.5 years. After replacement, the median follow‐up was 4.2 years (2.0–6.8 years) during which 64 patients (29%) died at a median interval of 2.7 years (1.0–4.2 years). Overall, the annual mortality rate was 6.8%, yielding an overall mortality rate of 9% and 28%, at 1 and 5 years, respectively. Mortality was not different between the different implant periods (*p* = .56).

**Table 1:** Patient characteristics at initial implantation and replacement (*n* = 218)

Variable	Implantation	Replacement	*p* Value
Age (years)	65 (58–71)	70 (62–75)	<.001
Male gender	159 (73%)	–	–
Atrial fibrillation	65 (30%)	76 (35%)	<.001
Ischemic cardiomyopathy	94 (43%)	94 (43%)	1.00
Myocardial infarction	67 (31%)	67 (31%)	1.00
NYHA functional class			
I–II	61 (28%)	167 (77%)	<.001
III–IV	157 (72%)	51 (23%)	<.001
LVEF (%)	25 ± 6	36 ± 11	<.001
LVEF ≤ 35%	218 (100%)	122 (56%)	<.001
QRS duration (ms)	167 ± 24	–	–
Left bundle branch block	178 (81%)	–	–
Laboratory data			
Sodium (mmol/L)	140.0 ± 3.5	140.0 ± 2.9	.04
Hemoglobin (g/dl)	14.0 ± 1.7	13.6 ± 1.6	<.001
Creatinine (µmol/L)	92 (77–117)	102 (82–136)	<.001
Glomerular filtration rate (ml/min/1.73 m^2^)	71 (52–88)	61 (41–80)	<.001
Medical therapy			
Beta‐blocker	177 (81%)	192 (88%)	.006
ACEI/ARB	209 (96%)	204 (94%)	.38
MRA	89 (41%)	114 (52%)	<.001
Diuretic	176 (81%)	188 (86%)	.05
Digoxin	40 (18%)	54 (25%)	.007
Statin	136 (63%)	132 (61%)	.49

*Note*:  Continuous data are presented as mean ± *SD* or as median (interquartile range). Categorical data are presented as *n* (%).

Abbreviations: ACEI, angiotensin‐converting enzyme inhibitor; ARB, angiotensin receptor blocker; LVEF, left ventricular ejection fraction; MRA, mineralocorticoid receptor antagonist; NYHA, New York Heart Association.

The prevalence of comorbidities at implantation and replacement is presented in Table 2. Overall, an increase in non‐ICD indication‐related comorbidities was observed in 77 patients (35%). The proportion of patients with at least one non‐ICD indication‐related comorbidity increased between implantation and replacement; 63% of patients at implantation versus 76% at replacement (*p* < .001). Development of new CKD was observed in 19% of patients followed by diabetes mellitus (12%) and AF (11%). Following CRT implantation, renal function remained unchanged in 93 patients (43%) and worsening was observed in 98 patients (45%). In patients with CKD at replacement (*n* = 108), a worsening of CKD stage was observed in 77 patients (71%) whereas it remained unchanged in 25 patients (23%) when compared to their CKD stage at implantation (*p* < .001).

**Table 2:** Comorbidities at initial implantation and replacement (*n* = 218)

Comorbidity	Implantation	Replacement	*p* Value
Non‐ICD indication‐related comorbidity burden^a^			<.001
No comorbidities	80 (37%)	53 (24%)	
Comorbidity burden = 1	71 (32%)	55 (25%)	
Comorbidity burden ≥ 2	67 (31%)	110 (51%)	
Atrial fibrillation	65 (30%)	76 (35%)	<.001
Diabetes mellitus	49 (23%)	61 (28%)	<.001
Chronic obstructive pulmonary disease	24 (11%)	33 (15%)	.004
Cerebrovascular disease	21 (10%)	25 (12%)	.13
Peripheral vascular disease	11 (5%)	21 (10%)	.002
Cancer	8 (4%)	17 (8%)	.004
Anemia	38 (17%)	56 (26%)	.03
Chronic kidney disease (eGFR < 60 ml/min/1.73 m^2^)	78 (36%)	108 (50%)	<.001
Stages of chronic kidney disease			<.001
Stage 1, (eGFR ≥ 90 ml/min/1.73 m^2^)	46 (21%)	26 (12%)	
Stage 2, (eGFR 60–89 ml/min/1.73 m^2^)	94 (43%)	84 (39%)	
Stage 3A, (eGFR 45–59 ml/min/1.73 m^2^)	45 (21%)	43 (19%)	
Stage 3B, (eGFR 30–44 ml/min/1.73 m^2^)	29 (13%)	40 (18%)	
Stage 4, (eGFR 15–29 ml/min/1.73 m^2^)	2 (1%)	23 (11%)	
Stage 5, (eGFR < 15 ml/min/1.73 m^2^)	2 (1%)	2 (1%)	

Abbreviations: eGFR, estimating the glomerular filtration rate; ICD, implantable defibrillator.

^a^ Non‐ICD indication‐related comorbidity burden: atrial fibrillation, diabetes mellitus, chronic obstructive pulmonary disease, cerebrovascular disease, peripheral vascular disease, cancer, and chronic kidney disease.

**Figure 1:**
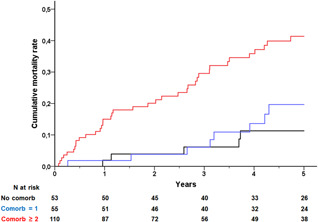
Cumulative mortality rates after cardiac resynchronization defibrillator (CRT‐D) replacement stratified by increasing comorbidity burden. Comorbidity was calculated non‐ICD indication‐related comorbidities present at the time of replacement, atrial fibrillation, diabetes mellitus, chronic pulmonary obstructive disease, cerebrovascular disease, peripheral vascular disease, chronic kidney disease, and cancer. No comorbidities (black line), one comorbidity (green line), and at least two comorbidities (red line). *p* Value for log‐rank less than .001. ICD, implantable defibrillator

Increasing comorbidity burden was associated with an increased risk of mortality (Figure 1). For patients without any non‐ICD indication‐related comorbidity, the 5‐year mortality rate was 11%. It was 20% in patients with one comorbidity, and 41% in patients with at least two comorbidities at the time of replacement. AF, anemia, and CKD, adjusted for age and LVEF at replacement and gender, were independently associated with increased risk of mortality postreplacement (Table 3).

**Table 3:** Individual univariate logistic regression fitted for each comorbidity adjusted for age and gender

Comorbidity	Events *N* of patients (%)	Incidence rate per 100 py (95% CI)	Odds ratio (95% CI)	*p* Value
Atrial fibrillation	28 (37%)	12.5 (8.7–18.2)	2.63 (1.35– 5.12)	.004
Ischemic cardiomyopathy	20 (30%)	8.3 (5.3–12.8)	1.37 (0.69– 2.69)	.37
Diabetes mellitus	17 (28%)	7.6 (4.7–12.2)	1.41 (0.70– 2.83)	.34
Chronic obstructive pulmonary disease	11 (33%)	11.0 (6.1–19.8)	1.67 (0.74– 3.80)	.22
Cerebrovascular disease	10 (40%)	13.4 (7.2–24.9)	2.08 (0.85– 5.14)	.11
Peripheral vascular disease	4 (19%)	6.1 (2.3–16.2)	0.63 (0.20– 2.00)	.43
Cancer	6 (35%)	9.5 (4.3–21.2)	1.62 (0.55– 4.75)	.38
Anemia	22 (39%)	12.1 (7.9–18.3)	2.42 (1.21– 4.86)	.01
Chronic kidney disease (GFR < 60 ml/min/1.73 m^2^)	35 (32%)	10.3 (7.4–14.4)	2.55 (1.24– 5.25)	.01

Abbreviations: CI, confidence interval; GFR, glomerular filtration rate; PY, person‐years.

Appropriate ICD shock before replacement occurred in 27 patients (12%) yielding an incidence rate of 2.6 per 100 person‐years no difference between the different implant groups (*p* = .48). Appropriate ICD shocks before replacement was associated with an increased risk for mortality after replacement (OR: 9.6, 95% CI: 4.0–23.2; *p* < .001). Considering appropriate ATP before replacement which occurred in 40 patients (18%), no association with mortality after replacement was found (*p* = .50).

Using age, gender, LVEF, AF, anemia, CKD, and history of appropriate ICD shocks at replacement, a risk score was developed to predict mortality after replacement of CRT‐D (Table SI).

$$\begin{array}{lll} \text{DARC}\;\text{risk}\;\text{score} & = & 0.257\times (\text{Age}50)+0.471\times (\text{Gender})\\ & & +\;0.989\times (\text{LVEF}35)+0.512\times (\text{AF})+0.745\\ & & \times \;(\text{Anemia})+0.382\times (\text{eGFR}60)+1.897\\ & & \times \;(\text{appropriate}\;\text{ICD}\;\text{shock}), \end{array}$$

where Age50 = per decade increase of age in patients with age greater than 50 years at replacement. In patients with age ≤ 50 years, the score associated with age is 0; anemia = serum level of hemoglobin less than 12 g/dl (female) or less than 13 g/dl (male), 1 if present, otherwise 0; eGFR 60 = estimated GFR per 15 ml/min/1.73 m^2 ^in patients with eGFR less than 60 ml/min/1.73 m^2^ at replacement. In patients with eGFR ≥ 60 ml/min/1.73 m^2^, the score associated with eGFR is 0; male gender = 1, female gender = 0; LVEF35 = 1 when LVEF ≤ 35% at replacement. In patients with LVEF > 35%, the score is 0; AF, ICD shock = 1 if present between implant and replacement, otherwise 0.

**Figure 2:**
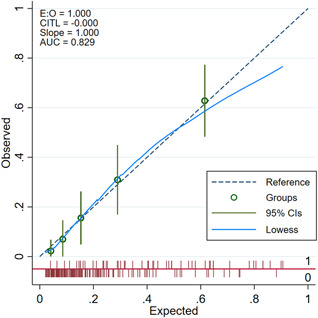
Calibration plot of the predicted probability against the observed proportion of mortality. Circles represent quintiles of subjects grouped by similar predicted risk with 95% Cl. The distribution of subjects is indicated with spikes at the bottom of the graph, stratified by mortality (death above the *X*‐axis, survivors below the *X*‐axis). AUC, area under the curve; CI, confidence interval

Bootstrapping the multivariate logistic regression analysis in 1000 simulated samples demonstrated identical *p* values and comparable coverage of the associated 95% CIs for the odds ratios. Model discrimination as assessed by the *C*‐statistic was 0.829 (95% CI: 0.767–0.891; *p* < .001). The calibration was good (Figure 2).

**Figure 3:**
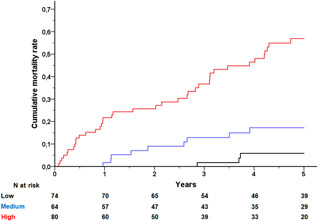
Cumulative mortality rates after cardiac resynchronization defibrillator (CRT‐D) replacement stratified by risk group according to DARC risk score. Low risk (black line), medium risk (blue line), and high risk (red line). *p* Value for log‐rank less than .001. DARC, death after replacement of CRT‐D

The median DARC risk score for all patients was 2.0 (1.2–3.1). The median DARC risk score among survivors and nonsurvivors was highly different (1.8 vs. 3.4; *p* < .001). The score values were rounded to the first decimal and patients were stratified into three risk groups (low, score 0–1.5; medium, score 1.5–2.5; high, score > 2.5). The mortality rates stratified by three risk groups are presented in Figure 3. At 1‐year postreplacement, mortality ranged from 0% (low risk), 2% (medium risk) to 22% (high risk). At 5‐year postreplacement, mortality was 57% in the high‐risk group versus 6% (low risk) and 17% (medium risk). How to calculate the DARC risk score and the assignment of mortality risk are presented in Supporting Information Materials Online.

**Figure 4:**
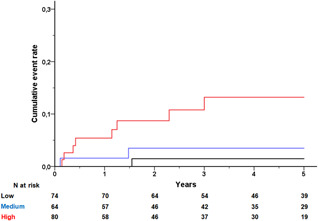
Cumulative appropriate shock rates after cardiac resynchronization defibrillator (CRT‐D) replacement stratified by risk group according to DARC risk score. Low risk (black line), medium risk (blue line), and high risk (red line). *p* Value for log‐rank .015. DARC, death after replacement of CRT‐D

After generator replacement, 11 patients (5%) received appropriate ICD shocks, yielding an incidence rate of 1.5 per 100‐person years. Appropriate ICD shock rate was not different between patients with LVEF ≤ 35% versus those with LVEF > 35% (*p* = .22). The cumulative appropriate shock rates stratified by three risk groups are presented in Figure 4. At 5‐year postreplacement, appropriate shock rate ranged from 2% (low risk), 4% (medium risk) to 13% (high risk). In the high‐risk group, 85% of the deceased patients experienced no appropriate ICD shocks after replacement.

## 4 DISCUSSION

In this study, we present the evolution of comorbidities and association with mortality after CRT‐D replacement in primary prevention patients from two international tertiary centers. Furthermore, we developed a dedicated mortality risk tool, the DARC risk score, for patients at the time of CRT‐D replacement. In the present study, 29% of patients died at a median interval of 2.7 years after replacement. We demonstrate that an increasing non‐ICD indication‐related comorbidity burden has a cumulative effect on mortality. The presence of atrial AF and anemia, level of CKD, LVEF, and past appropriate ICD shocks before replacement was highly predictive of mortality after elective replacement. In the high‐risk group, the majority of patients die without appropriate ICD shock therapy after replacement.

A few studies with heterogeneous study populations reported mortality rates following device replacement. A substudy of the REPLACE Registry reported an overall 6 months all‐cause mortality rate of 4%.^6^ The study by Kramer et al.^7^ reported a 1‐year mortality rate of 9.8% following ICD and CRT‐D replacement. The reported 5‐year mortality rates ranged from 25% to 41% in previous studies.^7^, ^8^, ^9^, ^13^ We found similar overall mortality rates following CRT‐D replacement in a patient group with primary preventive ICD implant, 9% at 1‐year and 28% at 5‐year follow‐up.

Several studies have consistently demonstrated variables such as age, AF, HF severity as well as noncardiac comorbidities such as CKD, COPD, CVA, and PVD to constitute predictors of mortality after initial implant.^14^, ^15^, ^16^, ^17^ Importantly, extensive concomitant noncardiac comorbidity has been associated with increased mortality risk. Ruwald et al.^17^ investigated a mixed cohort of primary and secondary ICDs and CRT‐Ds and found a greater than 50% mortality risk at 4 years in patients with comorbidity burden ≥3.

When considering replacement, cardiovascular morbidity and noncardiac comorbidity between implant and replacement might have evolved. The study by Kini et al.^18^ found among patients with a primary prevention ICD and CRT‐D, a significantly higher prevalence of comorbidities such as CKD, AF, and diabetes at the time of generator replacement compared to initial implant. More recently, Wuest et al.^8^ investigated a mixed cohort of primary and secondary ICDs (and CRT‐Ds) and found a decrease of GFR > 20 ml/min/1.73 m^2^ in 30% of patients at replacement. In the present study, an increased comorbidity burden was observed in 35% of patients. In a recent analysis of a nationwide cohort of patients with a primary prevention ICD (and CRT‐D) who underwent generator replacement, high mortality rates were found among those with ≥3 noncardiac comorbidities, showing 1‐ and 4‐year mortality rate of 36% and 73%, respectively. Our data are ancillary of this finding, high age and increased comorbidity burden at the time of replacement are associated with increased mortality risk.

Discussion on benefits of generator replacement should also consider appropriate ICD shocks delivered by the first generator in addition to advanced age and comorbidity burden. Several studies reported a lower risk of appropriate ICD therapy in primary prevention CRT‐D patients in whom LVEF recovery was observed during follow‐up.^19^, ^20^, ^21^, ^22^ In our study, we found no association between appropriate shocks and LVEF recovery. Irrespective of LVEF recovery, 5% of patients received appropriate ICD shocks following replacement with an incidence rate of 1.5%, which is lower than previously reported incidence rates. However, when comparing studies on ICD therapy and LVEF recovery, several aspects have to be considered. In our study, only appropriate ICD shocks within the VF zone (being potentially life‐threatening) in patients with a CRT‐D were considered. Other studies have a mixed population of patients with ICD or CRT‐D or event rates based on any ICD therapy (ATP or shock). The meta‐analysis by Chatterjee et al.^22^ demonstrated that patients with LVEF recovery and those with primary prevention indications appear to be at the lowest risk for ventricular arrhythmias due to CRT. A protective effect of CRT may explain the lower rate of appropriate shocks. Of note, the majority of the patients who died experienced no appropriate ICD shocks after replacement. Previous studies have shown also reduced ICD benefit which was associated with increased comorbidity and advanced age.^9^, ^23^

Based on the aforementioned studies, it is unknown how to best manage primary prevention patients with a CRT‐D regarding generator replacement. The decision to downgrade a CRT‐D to a CRT pacemaker (CRT‐P) is challenging and should take into consideration several aspects, such as the age of the patient, comorbidity burden, risk of appropriate ICD therapy, and patient preferences. We developed a simple risk stratification tool incorporating age, gender, LVEF, comorbidity, and prior appropriate ICD shock therapy. This tool identifies a subgroup of patients at very high risk of mortality which may assist in shared decision‐making between patient and physician whether the patient's status and preference merit the same type of device, a CRT‐D, or a new one without defibrillator therapy, a CRT‐P.

## 5 LIMITATIONS

Although the analysis was retrospective, data in both registries, including mortality and appropriate ICD shocks were all collected prospectively. The study cohort included patients over a 12‐year period, during which guidelines for the implantation of defibrillators and treatment of HF changed. In the same period, the programming of devices with respect to the detection and treatment of ventricular arrhythmias changed. We accounted for this by defining three groups according to the date of the implant. Although the DARC risk score accurately identified patients at high risk for mortality after replacement, external validation could not be performed. Internal validation has been performed by bootstrap analysis. We encourage further studies to validate our findings of the DARC risk score in a larger cohort. In addition, the study included only patients who underwent CRT‐D replacement. Patients who did not undergo CRT‐D replacement and those who were downgraded to a CRT‐P were not included in the current analysis.

## 6 CONCLUSION

In this real‐life cohort of primary prevention CRT‐D patients, we observed a significant increase in comorbidity burden between initial implantation and elective generator replacement. A high comorbidity burden was associated with increased mortality after replacement. Age, gender, LVEF, comorbidity, such as CKD and anemia, and prior appropriate ICD shock therapy were identified as contributors to mortality after generator replacement. A simple risk score accurately predicts 5‐year mortality after replacement, as patients with a risk score of greater than 2.5 are at high risk of dying despite ICD protection. In the future, clinical trials are necessary to evaluate the clinical benefits of CRT‐D replacement or downgrade to CRT‐P.

## Supporting information

Supporting information.

Supporting information.

## Data Availability

The data that support the findings of this study are available from the corresponding author upon reasonable request.
