# Supplementary material for: Reassessment of clinical variables in cardiac resynchronization defibrillator patients at the time of first replacement: Death after replacement of CRT (DARC) score
Source: J Cardiovasc Electrophysiol. 2021 Apr 30;32(6):1687–94. doi: 10.1111/jce.15031 (PMC8251620; doi:10.1111/jce.15031)
Supplement: Supplementary file 1 — Supporting information. [file JCE-32-1687-s001.docx]

**Supplemental Table I** Model construction to predict mortality

|  | **Model 1** | | **Model 2** | | **Model 3** | | **Model 4** | | **Model 5** | |
| --- | --- | --- | --- | --- | --- | --- | --- | --- | --- | --- |
| **Variable** | **β** | ***P*-value** | **β** | ***P*-value** | **β** | ***P*-value** | **β** | ***P*-value** | **β** | ***P*-value** |
| Age 50 (per 10 years) * | 0.023 | 0.263 | 0.016 | 0.434 | 0.017 | 0.411 | 0.027 | 0.229 | 0.028 | 0.219 |
| Male gender | 0.581 | 0.197 | 0.628 | 0.169 | 0.732 | 0.105 | 0.641 | 0.173 | 0.480 | 0.314 |
| LVEF ≤ 35% | 1.232 | 0.003 | 1.140 | 0.006 | 1.104 | 0.007 | 0.922 | 0.029 | 0.990 | 0.022 |
| Atrial fibrillation | 0.701 | 0.050 | 0.618 | 0.091 | 0.646 | 0.076 | 0.556 | 0.145 | 0.501 | 0.192 |
| Anemia | 0.688 | 0.078 | 0.515 | 0.195 | - | - | - | - | 0.745 | 0.075 |
| CKD | 0.466 | 0.245 | - | - | - | - | - | - | - | - |
| GFR (per 15 mL/min) ǂ | - | - | 0.524 | 0.013 | 0.607 | 0.003 | 0.502 | 0.018 | 0.374 | 0.095 |
| Appropriate ICD shock(s) | - | - | - | - | - | - | 1.796 | < 0.001 | 1.903 | < 0.001 |
| **Parameter** |  | |  | |  | |  | |  | |
| Goodness of fit |  | |  | |  | |  | |  | |
| Log LR chi-square | 35.63 | | 40.58 | | 38.93 | | 52.89 | | 56.03 | |
| AIC | 216 | | 210 | | 210 | | 198 | | 197 | |
| BIC | 239 | | 234 | | 230 | | 221 | | 224 | |
| Discrimination |  | |  | |  | |  | |  | |
| *C*-statistic | 0.762 | | 0.787 | | 0.786 | | 0.830 | | 0.829 | |
| CKD = chronic kidney disease; GFR = glomerular filtration rate; LVEF = left ventricular ejection fraction.  AIC = Akaike information criterion; BIC = Bayesian information criterion; LR = likelihood ratio.  * The β-coefficient represents the effect of age associated with 10 years change in patients with age > 50 years. In patients with age ≤ 50 years, the score associated with LVEF is 0.  ǂ The β-coefficient represents the effect of GFR associated with 15 mL/min change in patients with GFR < 60 mL/min/1.73 m^2^. | | | | | | | | | | |
