# Supplementary material for: Reassessment of clinical variables in cardiac resynchronization defibrillator patients at the time of first replacement: Death after replacement of CRT (DARC) score
Source: J Cardiovasc Electrophysiol. 2021 Apr 30;32(6):1687–94. doi: 10.1111/jce.15031 (PMC8251620; doi:10.1111/jce.15031)
Supplement: Supplementary file 2 — Supporting information. [file JCE-32-1687-s002.docx]

**Calculation of the DARC (Death After Replacement of CRT) score**

**Example 1:** Consider a 58-year old male patient with non-ischemic cardiomyopathy, the device reached the ERI status. No appropriate ICD shocks since implantation, no atrial fibrillation. Other measurements, LVEF 40%, creatinine of 184 µmol/L (GFR 34 ml/min) and hemoglobin 13.1 g/dL.

The DARC score is calculated by multiplying the β coefficient by the variable and summing the values. Binary predictors are coded as 1 if the characteristic is present and 0 when absent.

| **Predictor** | **β-coefficient** | **Factor** | **Applied example** | **Score** |
| --- | --- | --- | --- | --- |
| Age (per decade) * | 0.257 | (58-50)/10 | 0.257 * 0.8 | 0.206 |
| Male gender | 0.471 | 1 | 0.471 * 1 | 0.471 |
| LVEF | 0.989 | 0 | 0.989 * 0 | 0 |
| Atrial fibrillation | 0.512 | 0 | 0.512 * 0 | 0 |
| Anemia | 0.745 | 0 | 0.745 * 0 | 0 |
| GFR (per 15 mLmin) # | 0.382 | (60-34)/15 | 0.382 * 1.73 | 0.661 |
| Appropriate shock | 1.897 | 0 | 1.897 * 0 | 0 |
|  |  |  | **SUM** | 1.338 |
|  |  |  | **DARC score** | **1.3** |

* The β coefficient represents the effect of age associated with 10-year change in patients older than 50 years at time of replacement. If younger than 50 years, the score is set to 0.

# The β coefficient represents the effect of GFR associated with a 15 ml/min change in patients with GFR < 60 mL/min. In patients with GFR ≥ 60 mL/min, the score is set to 0. GFR should be estimated using the CKD-EPI formula.

**Example 2:** Consider a 69-year old female patient with non-ischemic cardiomyopathy, the device reached the ERI status. Since implant, she experienced appropriate ICD shocks and has atrial fibrillation. Other measurements, LVEF 35%, creatinine of 101 µmol/L (GFR 49 ml/min) and hemoglobin 10.9 g/dL.

| **Predictor** | **β-coefficient** | **Factor** | **Applied example** | **Score** |
| --- | --- | --- | --- | --- |
| Age (per decade) * | 0.257 | (69-50)/10 | 0.257 * 1.9 | 0.488 |
| Male gender | 0.471 | 0 | 0.471 * 0 | 0 |
| LVEF | 0.989 | 1 | 0.989 * 1 | 0.989 |
| Atrial fibrillation | 0.512 | 1 | 0.512 * 1 | 0.512 |
| Anemia | 0.745 | 1 | 0.745 * 1 | 0.745 |
| GFR (per 15 mLmin) # | 0.382 | (60-49)/15 | 0.382 * 0.73 | 0.280 |
| Appropriate shock | 1.897 | 0 | 1.897 * 0 | 0 |
|  |  |  | **SUM** | 3.014 |
|  |  |  | **DARC score** | **3.0** |

**Table Mortality risk and DARC score**

| **DARC risk group** | **DARC Score** |
| --- | --- |
| **Low** | **0 – 1.50** |
| **Medium** | **1.50 – 2.50** |
| **High** | **> 2.50** |
